# Antimicrobial and antivirulence function of cinnamaldehyde against *Streptococcus suis* type 2

**DOI:** 10.1128/spectrum.02561-24

**Published:** 2025-02-13

**Authors:** Lexin Zhu, Zhishu He, Mengqing Li, Jixin Xu, Wei Ding, Wenzhen Zeng, Xiaowu Jiang

**Affiliations:** 1 College of Medicine, Yichun University, Yichun, Jiangxi, China; 2 Jiangxi Provincial Key Laboratory of Active Component of Natural Drugs, Poster-Doctoral Research Center, Yichun, Jiangxi, China; Michigan State University, East Lansing, Michigan, USA

**Keywords:** cinnamaldehyde, *Streptococcus suis *type 2, antimicrobial, virulence, suilysin

## Abstract

*Streptococcus suis* type 2 (SS2) is an important zoonotic pathogen for swine and humans. The increasing prevalence of antimicrobial resistance in *S. suis* isolates poses a threat to public health. This study investigated the antimicrobial activity and therapeutic potential of cinnamaldehyde (CA), a natural compound from cinnamon, against SS2. CA showed significant antimicrobial activity with a minimal inhibition concentration of 0.25 µg/mL and prolonged post-antibiotic effect of over 7 h in SS2. Increased bacterial cell membrane permeability and blocked protein synthesis of SS2 were observed after being treated with CA. CA could effectively prevent biofilm formation. CA treatment reduced the crucial virulence factor of suilysin expression and secretion in SS2 cells through a probable interaction with the suilysin protein. CA treatment could prominently alleviate both epithelial HEp-2 and phagocytic RAW264.7 cell cytotoxicity induced by SS2. The pathogenic SS2 strain was attenuated by CA, as demonstrated by the diminished adherence in HEp-2 cells, increased clearance by RAW264.7 and mice whole blood, and improved survival rate in a mouse infection model. CA possessed therapeutic efficacy since the CA treatment exhibited a 50% improvement in mouse survival rate, which surpassed the traditional ampicillin therapy control group. Alleviated clinical symptoms and histopathological phenotypes, with reduced bacterial burden in mouse organs after CA treatment, were examined. Overall, this study identified cinnamaldehyde as a novel antibacterial compound against SS2 with potential therapeutic protective effects, offering an alternative drug for controlling SS2 prevalence and infection.

**IMPORTANCE:**

Widespread infections caused by *Streptococcus suis* type 2 (SS2) have garnered significant attention in the realm of public health due to their zoonotic nature. In recent years, antimicrobial resistance phenotypes in SS2 have emerged and intensified within the context of animal husbandry. Herbal compounds and medicinal plants are increasingly recognized as promising therapeutic alternatives for mitigating or addressing the challenges posed by antimicrobial resistance. The aim of this present study was to explore a novel compound of cinnamaldehyde, which obtained significant antimicrobial activity and potential therapeutic protective effect against SS2 infection. The research has made an innovative discovery that the bactericidal effect of cinnamaldehyde is associated with its antivirulence strategies, such as targeting the key virulence factors of SS2 and countering the bacterial infection process.

## INTRODUCTION

*Streptococcus suis* is a common zoonotic agent capable of causing serious pathogenic disorders such as arthritis, endocarditis, septicemia, streptococcal toxic shock-like syndrome (STSLS), and meningitis (1, 2). Currently, emerging/re-emerging *S. suis* can be recognized and consisted of at least 29 serotypes, distinguished by their divergent capsular polysaccharide antigens. *S. suis* serotype 2 (SS2) is the most prevalent and virulent isolate in pigs and humans (3). *S. suis* infections induce substantial economic losses in pig-producing countries globally and lead to intensive hazards for individuals in close proximity to pigs or pork products (4–6). Three large-scale outbreaks of SS2 infection among humans in Sichuan, Jiangsu, and Guangxi provinces have been reported in China, respectively (7, 8). The available strategies for controlling *S. suis* infection still depend on veterinary vaccines and effective antibiotics. However, only partial or serotype-specific protection is induced by vaccination, and the prophylactic or indiscriminate application of antimicrobials in livestock production hampers the clearance program for *S. suis* occurrence (4, 9, 10). Particular strains of *S. suis*, which were found to harbor antimicrobial resistance (AMR) genes and mobile genetic elements, are now considered as a newly widespread superbug, attributed to the rising prevalence of multidrug resistances (11). The highest AMR rates for tetracyclines, lincosamides, macrolides, and aminoglycosides have been reported in *S. suis* (11, 12). A study from northeast India revealed that the resistance pattern of *S. suis* isolates formed 16 resistance groups (R1 to R16), with 93.54% of the isolates exhibiting multidrug resistance (resistant to three or more antimicrobial agents) (13). In China, over 80% of 314 isolates collected in Jiangxi between 2017 and 2019 were resistant to penicillin, minocycline, and chloramphenicol (14). These epidemiological investigations posed a global public health concern related to One Health and highlighted an urgent need for developing alternative strategies to mitigate the societal impact stemming from *S. suis* infections.

Natural plant products, mainly phytochemicals and their derivatives, have historically served as a primary source of effective therapeutic substances upon the long-standing medical practices in our traditional medical systems (15, 16). A variety of functional extracts and active ingredients from medicinal plants have been explored as herbal antimicrobials that can combat pathogen infection (17). Cinnamaldehyde (CA), a naturally predominant active constituent derived from cinnamon oil in the stem bark and leaves of *Cinnamomum* genus, is widely available and used in perfumes, cosmetics, food additives, and the pharmaceutical industries (18). CA is a promising natural preservative that possesses antimicrobial and antivirulence properties against various harmful isolates of bacteria, yeasts, filamentous molds, and dermatophytes (19, 20). CA is non-toxic and safe for inducing bactericidal effects and inhibiting the production of microbial secondary metabolites (21, 22). AMR pathogens, including *Enterococcus faecalis*, *Proteus mirabilis*, *Acinetobacter baumannii*, *Aeromonas hydrophila*, *Staphylococcus aureus*, *Pseudomonas aeruginosa*, and *Listeria monocytogenes*, have been reported to be susceptible to CA exposure and effectively eradicated by CA through potential mechanisms, such as damaging the cell membrane, altering the lipid profile, inhibiting ATPases, disrupting cell division, affecting membrane porins, impeding motility, and preventing biofilm formation (23–30). However, the antimicrobial activity of CA against SS2 remains unclear.

Hence, in this study, the inhibitory efficacy of CA against SS2 and the effects of CA on microbial phenotypic changes, virulence alteration, and potential therapeutic function in mice were assessed. This study may contribute to the development of a medicinal compound of CA as one of the newly proposed alternative antimicrobial agents for controlling *S. suis* infection.

## MATERIALS AND METHODS

### Bacterial strain and reagent

The *S. suis* type 2 strain of HA9801, which was isolated from the outbreak of SS2 in 1998 in China, was provided by Professor Weihuan Fang from the Laboratory of Animal Preventative Medicine, Zhejiang University, China. SS2 strain was cultured in brain heart infusion (BHI, OXOID, UK) at 37°C with gentle agitation at a speed of shaking at 120 rpm. CA with a purity of over 98% was procured from Shanghai Macklin Biochemical Technology Co.

### Antibacterial susceptibility test

The minimum inhibitory concentration (MIC) and minimum bactericidal concentration (MBC) were determined using the conventional microtiter broth dilution method in accordance with the guidelines established by the Clinical and Laboratory Standards Institute. Overnight cultures of SS2 were centrifuged, rinsed twice with phosphate Buffered Saline (PBS), and then adjusted to an OD_595 nm_ of 0.3. CA was serially diluted twofold in 96-well flat-bottom plates, and an equal volume of 100 µL SS2 suspension at a 1,000-fold dilution was added to each well. The mixture was incubated aerobically at 37°C for 20–24 h. SS2 inoculation with CA-free broth and BHI culture without SS2 were used as the controls. The lowest CA concentration with invisible bacterial growth and no viable colonies subcultured on agar plates were deﬁned as MIC and MBC, respectively. All experiments were performed in triplicate.

### Growth kinetics

The bacterial growth assay of SS2 was performed as described method (31). CA was diluted at concentrations of 1/2×, 1/4×, 1/8×, and 1/16× MIC and cocultured with SS2 in a sterile microbial culture plate at 37°C for 48 h. SS2 without CA treatment was used as a control. The absorbance at OD_595 nm_ was measured every 2 h intervals. The surviving bacteria in the different groups were counted by serial dilution and plating method. This assay was performed three times in triplicate wells.

### Post-antibiotic effect (PAE)

PAEs were examined as previously described with minor modification (32). Overnight SS2 cultures in the exponential growth phase were collected via centrifugation, suspended in BHI broth, and adjusted to a starting inoculum of 10^7^ colony-forming units (CFU/mL). The SS2 suspension was then exposed to 0.25 µg/mL of CA at 37°C. Tetracycline treatment (160 µg/mL) and BHI broth free of any additives were synchronously performed as controls. After 2 h of incubation, the mixtures were centrifuged to remove bacterial supernatant in each group. The bacterial cells were washed three times with sterile PBS to eliminate the tested compounds and then resuspended in fresh BHI medium. All cultures in the post-antibiotic phase were incubated at 37°C for an additional 10 h. Viable SS2 colonies in each group were counted every 2 h in the BHI plates, and the dynamic changes of CFU in these groups were drawn. PAEs were determined by measuring the defined formula: *PAE* = *T* − *C*, where *T* represents the interval time required for the viable counts of the antibiotic-exposed cultures to increase by one log10 above the counts observed immediately after washing, and *C* represents the corresponding duration (h) for the unexposed BHI control group.

### Bacterial total protein synthesis and cell membrane permeability

Total bacterial protein synthesis was evaluated as described previously (33). Briefly, fresh SS2 bacterial cells (10^6^ CFU/mL) were suspended in BHI broth containing 1/4 MIC of CA (0.0625 µg/mL), and the mixtures were incubated at 37°C for 4 h and 8 h, respectively. SS2 incubated with BHI only was also included as a control group. The samples were centrifuged at specified time points to obtain the SS2 precipitate. The whole bacterial cell proteins of SS2 were harvested by using membrane protein extraction buffer and further disrupted with Precellys Homogenizer as previously described (34). SDS-PAGE and protein density quantification analysis with ImageJ software (ImageJ 1.5, NIH) were conducted to detect total bacterial proteins.

Late logarithmic SS2 cultures were centrifuged, washed three times with sterile PBS, and resuspended to adjust the initial SS2 count to 10^8^ CFU/mL in sterile PBS. CA at 0.25 µg/mL was added to the solution and processed into a static culture for 0, 2, 4, 6, and 8 h at 37°C. The effect of CA on bacterial cell membrane leakage was assessed by collecting the supernatants in each group and then determining the concentration of genomic DNA and protein contents by using a spectrophotometer (NanoDrop2000, Thermo) as described previously (35).

### Biofilm formation and eradication assays

Biofilm detection was performed by crystal violet staining, according to a previously described method (28). Briefly, the SS2 strain was cocultured with CA at 1/4×, 1/8×, and 1/16× MIC or BHI only in a ﬂat-bottom 96-well microplate for 96 h. After PBS washing for three times and fixing by methanol, the bacterial biofilm was treated with 1% crystal violet (wt/vol) and examined by microscopy. After crystal violet stain release by adding 95% ethanol, the absorbance at OD_595 nm_ in solutions was measured to quantify the biofilm formation capability. Supra-inhibitory concentrations of CA ranging from 4× to 1× MIC were applied to detect the destructive effect for preformed SS2 biofilm after 96 h incubation. The eradication process with CA was continued for 4 h at 37°C and further analyzed as the aforementioned biofilm detection method. The data were collected from three replicates and repeated three times.

### Hemolytic activity

The SS2 cultures treated with CA (varied from MBC and 1×, 1/2×, 1/4×, 1/8× MIC) or without CA (BHI only) for 16 h at 37°C were centrifuged to collect the supernatants. The hemolytic activity was measured as previously described (31). Briefly, equal volumes of 500 µL supernatant were added to 5% sheep red blood cells in PBS, incubated for 2 h at 37°C with 5% CO_2_, and centrifuged at 1,000 rpm for 10 min. Subsequently, 200 µL aliquots of the mixture supernatant were carefully harvested and measured at OD_540nm_ using a spectrophotometer microplate reader (Molecular Devices, USA). Triplicate repeat samples were used in this assay, and erythrocytes treated with 0.2% Triton-100 served as a 100% lysis control.

Probable interaction between CA and suilysin by *in silico* molecular docking analysis was performed by using the protein-ligand docking software AutodockVina 1.2.2 (36). The chemical structure of CA was acquired from PubChem, whereas the crystal structure of suilysin (PDB ID: 3HVN) was retrieved from the Protein Data Bank in the Swiss model.

### Virulence genes expression

Total RNA extractions from mid-logarithmic SS2 cultures processed with CA (0.0625 µg/mL) at 37°C for 16 h were purified and reverse-transcribed into cDNA following the instructions of the bacterial RNA isolation and cDNA synthesis reaction kit (Transgen, China), respectively. BHI cultured SS2 without CA treatment was performed as a control. Real-time qPCR reaction was employed to quantify the relative mRNA changes for 10 virulence factors by using the SYBR green detection method, which was conducted on the CFX Connect qPCR system (Bio-Rad, USA) as previously reported (37, 38), according to the primers listed in Table 1.

**TABLE 1:** Real-time quantitative PCR primer sequences

Targeted genes	Primers sequence
*Sly*	TGATGAACCAGAATCTCCAAGCAAG
	GTCTTGATACTCAGCATTGCCACTA
*Fbps*	AAGGTTTGGGTCGGGATA
	CAGAGCAGCATAGGATTTGT
*TGase*	AATCATGTAGTTACGCTCCG
	TACAGGGAATAAGCATCAGC
*enolase*	GACGTTCGTGATCAACAAGC
	CGCAACAGCGATAGAAACAC
*sodA*	TGGACGGACATTGCGGTAG
	TCGTTTCGGTTCAGGTTGG
*ssnA*	TCAGACGCTAAAGTCCAACG
	GAGGAAGCCGACGCGAATA
*SrtA*	TCAGTGGGATGCACAACG
	GCTCCATACATGAGCGAAG
*Rgg*	GAAGAGTTTGGCGGAGGT
	ATCCTGACTGGCATACACG
*neuB*	ACAACCGAGTATCCAACCC
	CGCTTTATGATCTGGTCCTT
*covR*	GTTGCCAGCAGATCATACT
	TGAAGCCCTTTGTCATTAG
*16s rRNA*	GTTGCGAACGGGTGAGTAA
	TCTCAGGTCGGCTATGTATC

Bacterial cultures with the above-mentioned treatment were subjected to supernatants collection and secreted proteins preparation by the trichloroacetic acid/acetone precipitation method as described previously (31). Western blotting analysis for suilysin or TGase was conducted, and protein profile in grayscale was quantified by ImageJ software. Protein detection was performed by using a chemiluminescent imaging system (ChemiDoc MP, Bio-Rad) with an Ultra-High Sensitivity ECL kit (GLPBIO, Montclair, CA, USA).

### *In vitro* cellular infection assays

The murine macrophage cell line RAW264.7 (ATCC, TIB-71) and human laryngeal epithelial HEp-2 (ATCC, CCL23) cells were cultured to evaluate SS2 cytotoxicity to host cells upon CA treatment. RAW264.7 or HEp-2 cells, cultured in 96-cell plate with a confluence of over 90%, were washed, incubated with CA at 1/2× and 1/4× MIC suspended in Dulbecco's Modified Eagle Medium (DMEM), and then infected with SS2 strain (‌multiplicity of infection, MOI = 100) for 4 h at 37°C with 5% CO_2_. DMEM or SS2 infection alone was synchronously conducted as negative and positive controls, respectively. Cell cytotoxicity was observed by immuno-ﬂuorescent microscopy with a LIVE/DEAD staining kit (Invitrogen, Eugene, OR) and determined using a lactate dehydrogenase (LDH) activity kit (Beyotime, China) as previously described (37). The safety and cytotoxicity evaluation of CA itself was performed as described above in a pilot assay.

Adherence to epithelial HEp-2 cells and phagocyte clearance by RAW264.7 macrophage cells were examined as previously reported methods (39). Overnight CA (1/2× and 1/4× MIC) cocultured SS2 suspension were centrifuged, washed twice by PBS, suspended in PBS, and adjusted to OD_595 nm_ as 0.4 consistently. Fifty microliters of each SS2 suspension was added to HEp-2 or RAW264.724 in 24-well plates and incubated at 37°C for indicated hours with 5% CO_2_. The SS2 culture without CA treatment was used as a control. Following PBS washing for four times, SS2 adhered to HEp-2 were lysed by sterile ddH_2_O and plated to BHI agar to count at proper dilutions. The intracellular viability of SS2 within RAW264.7 cells was performed similarly to the adhesion assay except that SS2 adhered cells encountered another 1 h incubation with gentamicin (300 µg/mL) before the final lysis step. Relative adhesion rates and viable SS2 counts after phagocytosis in the CA-treated or untreated groups were analyzed to assess the effect of CA on SS2 pathogenesis. All experiments were conducted in triplicate and were repeated on three separate occasions.

### *Ex vivo* viability within mice blood

The survival capability of the mouse whole blood was determined as previously described (40). The mid-exponential phase of SS2 cultured in BHI medium containing CA (0.0625 µg/mL) was adjusted to OD_595 nm_ at 0.3, and 100 µL of the SS2 suspension was inoculated into 900 µL of anti-coagulant fresh whole blood collected from clinically healthy mice (SLAC, China). SS2 cells cultured with BHI alone served as the control group. The mixtures were incubated for 1 h at 37°C, and viable SS2 numbers were counted by the serial dilution method on BHI agar plates.

### *In vivo* pathogenicity and anti-infection assays in mouse

Briefly, SS2 inoculates, either pre-incubated with CA (0.0625 µg/mL) or without CA for 12 h at 37°C, were collected and adjusted to an SS2 inoculation of approximately 5 × 10^8^ CFU/mL in PBS. Twenty-four female 6-week-old BALB/c mice (SLAC, China) were randomly assigned to three groups with eight mice in each, and subjected to SS2 suspension inoculation via an intraperitoneal approach. Sterile PBS was used as the negative control. Clinical symptoms and mouse mortality were monitored and recorded everyday within 1 week post-infection. To assess bacterial dissemination and SS2 load in organs, mice orbital blood with triplicate repeat after the aforementioned SS2 challenge test were collected at 4 h and 8 h post-infection, and mice after 12 h post-infection were euthanized for the collection of heart, brain, spleen, kidney, and lung samples. The samples were then homogenized and subjected to plate counting of SS2 following established protocols, as previously reported (41, 42).

The anti-infection assay of CA was conducted as previously described method (43, 44). Forty 6-week-old BALB/c mice were randomly assigned to four groups with 10 mice in each, and were pre-injected intraperitoneally with 1 mL of lethal SS2 solution at 5 × 10^8^ CFU. After 2 h of infection, mice were then administered with either CA (5 mg/kg), conventional ampicillin (5 mg/kg), or vehicle control of 10 mM PBS via intraperitoneal injection. The interval period for each treatment was 6 h, and the survival rate in each group was monitored within 7 days post-infection. SS2 load in various tissues were determined according to the infection model protocol mentioned above, and further histopathological analysis was performed successively by organ immobilization in 4% paraformaldehyde, thin-section, and hematoxylin/eosin staining for microscopic observation.

### Statistical analysis

Unless otherwise stated, all continuous experimental results were presented as mean ± standard deviation. The statistical program within GraphPad Prism (version 7.0) was used for data comparison, and the signiﬁcances of differences were defined using unpaired Student’s two-tailed *t*-test in two independent groups, one-way analysis of variance in three or more groups with Fisher’s least significant difference test for multiple comparisons, and the log-rank test in survival curves with the threshold for significance as *P < 0.05 and **P < 0.01.

## RESULTS

### Antimicrobial activity of cinnamaldehyde against SS2

**Fig 1:**
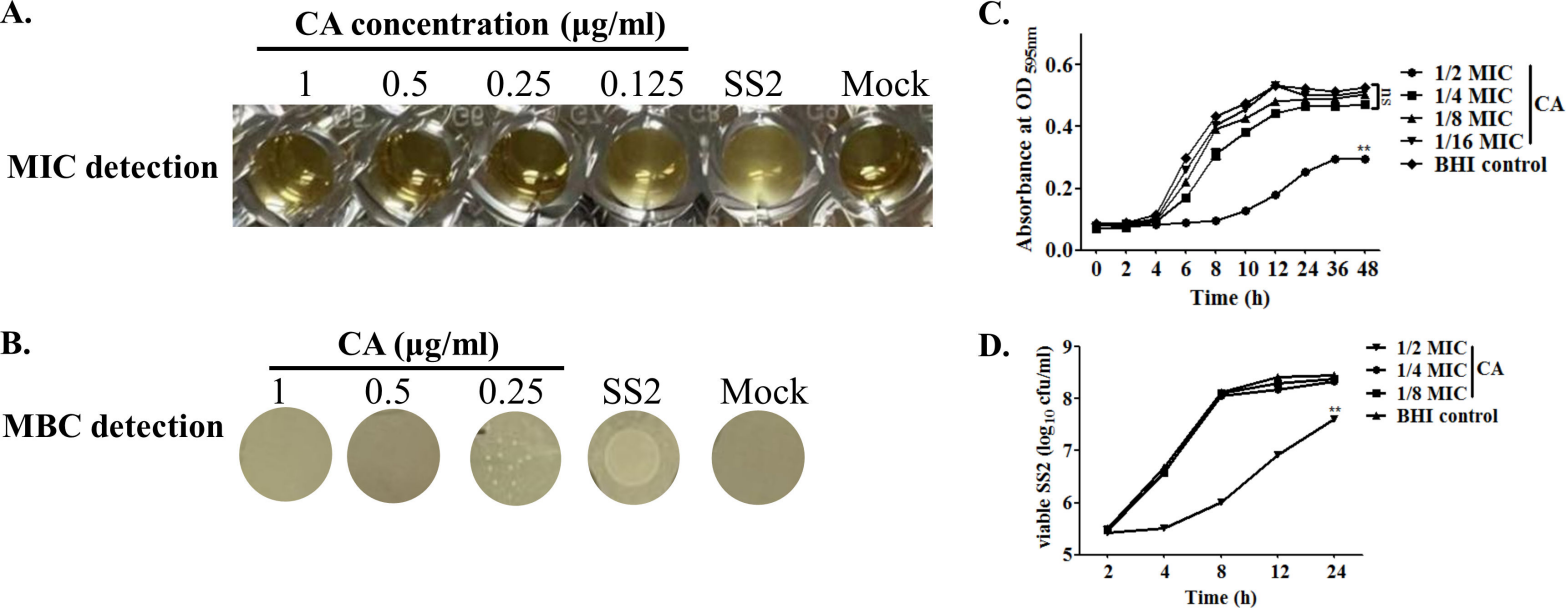
Antimicrobial activity of cinnamaldehyde against SS2. (**A and B)** MIC and MBC measurements for CA. (**C)** Growth curve for the SS2. The absorbance values at OD_595 nm_ were determined from CA treatment groups at different concentrations which cocultured with SS2 in a flat-bottom 96-well sterile microbial culture plate at 37°C for 48 h. (**D)** Bactericidal effects determined by BHI plate counting. The corresponding viable SS2 were counted in the CA-treated groups (1/2, 1/4, and 1/8 MIC) as described in C. SS2 cultured in the BHI medium was performed as a control group. Data in C and D were shown as mean ± SD for three replicate wells in one typical experiment. The experiments were all performed at least three times. The significances of “ns” and “**” was represented as *P* > 0.05 and *P* < 0.01.

The antimicrobial susceptibility test in Fig. 1A showed that the bacteriostatic effect of CA is concentration-dependent, with an MIC value of 0.25 µg/mL. The MBC of CA against SS2 was further determined as 0.5 µg/mL by BHI plate counting (Fig. 1B). The growth kinetics of SS2 cultured with CA at different concentrations showed that CA at less than 0.0625 µg/mL (i.e., groups in 1/4,1/8 and 1/16 MIC) could not inhibit bacterial growth, whereas CA at 1/2 MIC suppressed SS2 propagation and significantly reduced the viable counts of SS2 in the final observation time point as compared to the BHI control group (Fig. 1C and D).

### Cinnamaldehyde prolongs the PAE and modulates the cell membrane permeability and protein synthesis capacity on SS2

**Fig 2:**
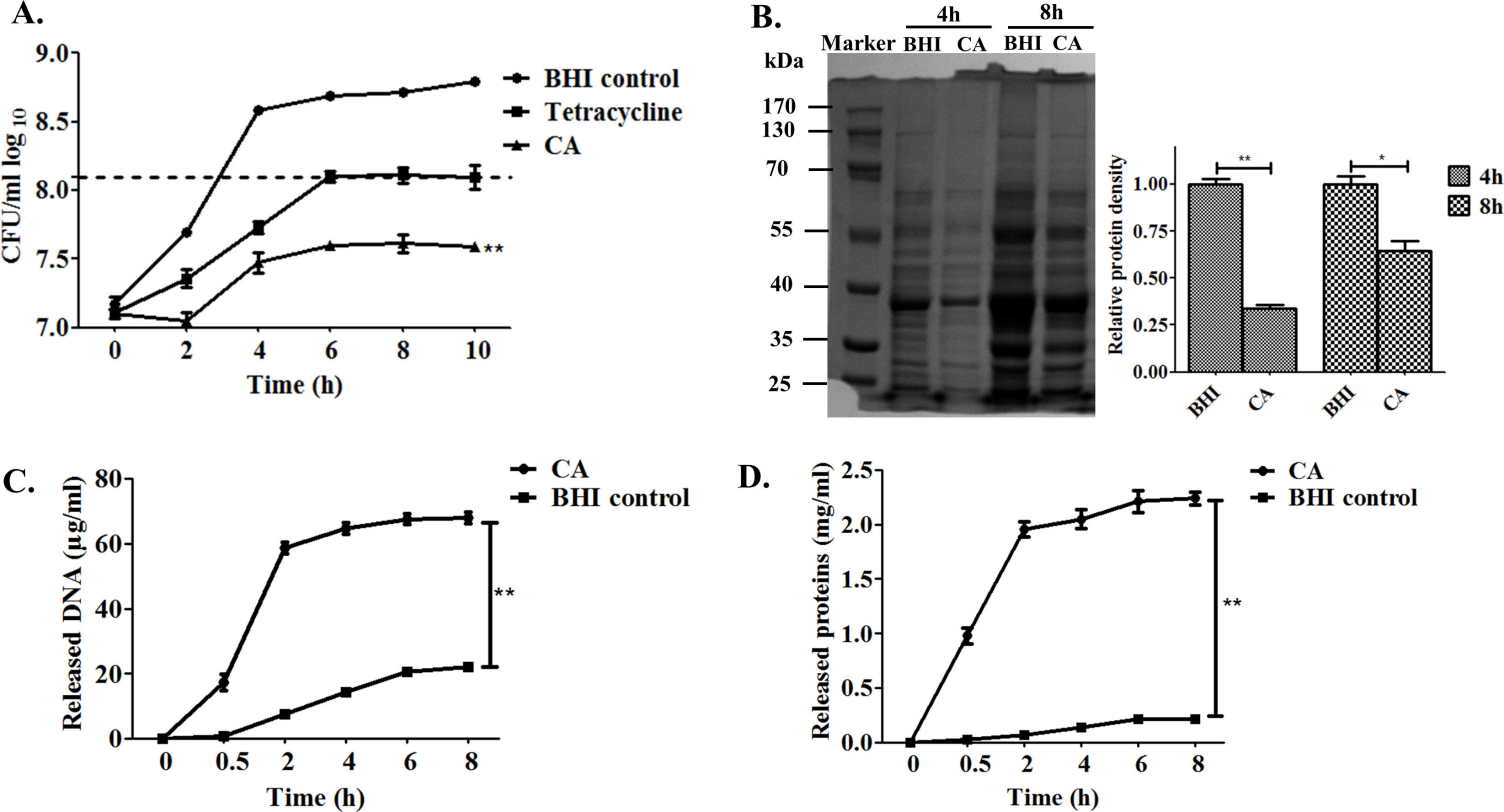
Cinnamaldehyde prolongs the PAE and modulates the cell membrane permeability and protein synthesis capacity of SS2. (**A)** Effect of cinnamaldehyde on PAE of SS2. The PAE kinetic indicated maintained suppression of SS2 growth following exposure to the pretreated 2 h of CA (0.25 µg/mL) at 37°C was determined. SS2 pretreated with tetracycline (160 µg/mL) and BHI broth only were conducted as positive and negative control, respectively. The dashed horizontal line represented as the one log_10_ increase by count number in the BHI control group.** (B)** SDS-PAGE for total protein analysis. SS2 suspension incubated with CA at 1/4 MIC and BHI broth control for 4 h or 8 h were harvested and subjected to bacterial whole cell protein extraction, protein electrophoresis, and further quantification analysis by ImageJ. (C and D) Kinetics of genome of DNA and cellular proteins leakage in SS2 supernatant after CA treatment at 0.25 µg/mL. All the experiments were repeated three times and significance labels “*” and “**” were represented as *P* < 0.05 and *P* <0.01.

The PAE refers to the persisting suppression impact of bacterial growth after a limited exposure to an antimicrobial agent. The period of time after which the antimicrobial has been eliminated, during which growth of the target bacteria is suppressed, was determined by time-killing kinetics based on a viability counting method. Interestingly, persistent inhibition effect of CA was shown by the lower viable SS2 count in Fig. 2A (*P* < 0.01). The interval time for SS2 in the BHI control culture to reach one log_10_ above the viable bacterial count was approximately 3 h. It took approximately 6 h for tetracycline to exhibit one log_10_ above the SS2 count increase, with a PAE value of 3 h in the tetracycline treatment (at 1× MIC) group. However, CA treatment (at 1 MIC) could maintain the bacteriostatic effect and control the SS2 growth within no more than one log_10_ above, thus displaying a notably longer PAE value of over 7 h against SS2 compared to the tetracycline control group.

The effects of CA on SS2 total protein synthesis and cell membrane permeability were detected by SDS-PAGE and quantified by measuring cellular content leakage. Significantly inhibited protein expression profiles of SS2 in CA treatment at a sub-inhibitory concentration of 1/4× MIC were indicated after 4 h and 8 h compared to the BHI control group (Fig. 2B). Furthermore, CA incubation with SS2 at 1× MIC would lead to obviously intracellular DNA genome and protein release in SS2 (*P* < 0.01) (Fig. 2C and D).

### Cinnamaldehyde prevents biofilm formation and disrupts preformed biofilm in SS2

**Fig 3:**
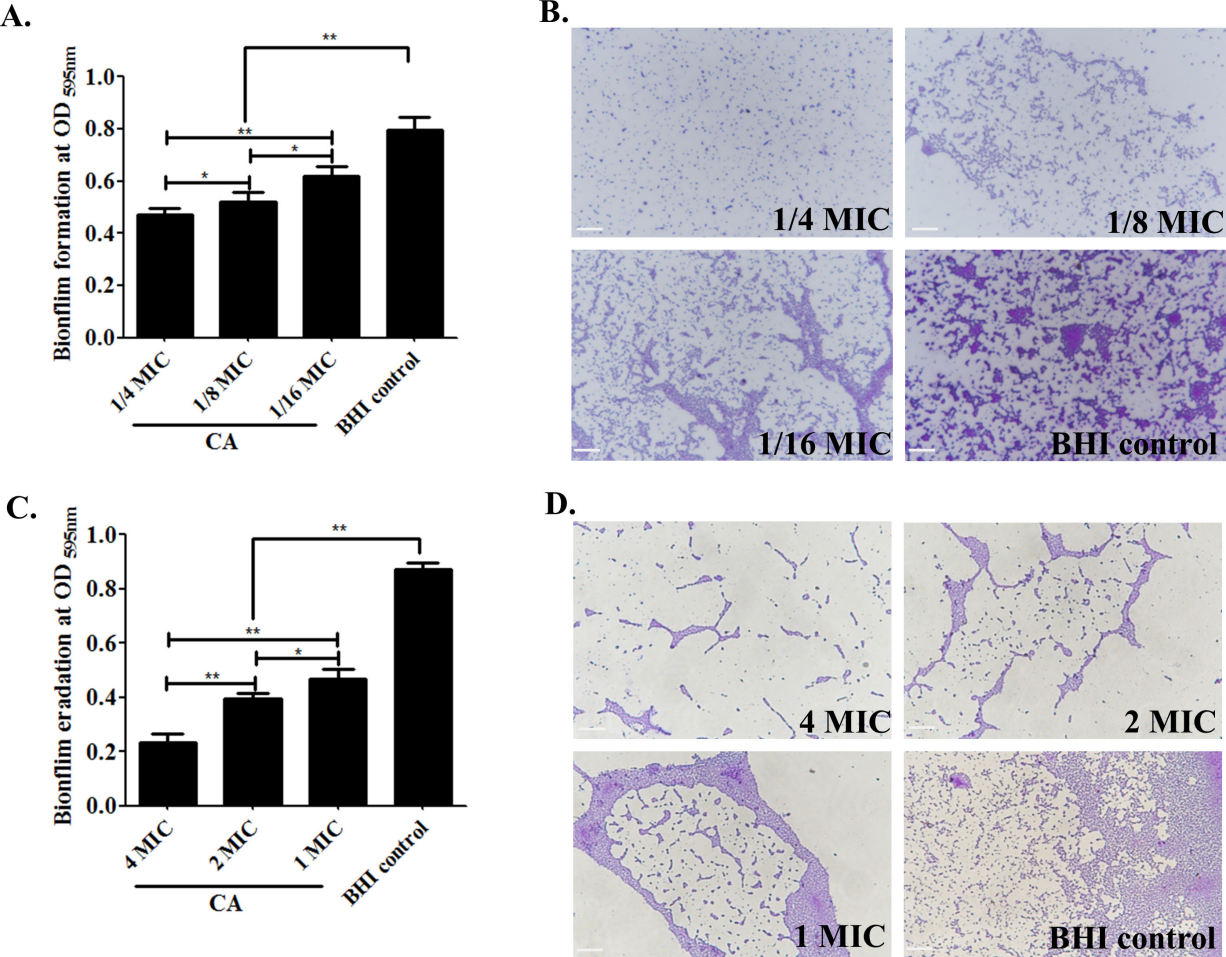
Cinnamaldehyde inhibits biofilm formation and disrupts preformed biofilm in SS2. (**A and B)** Effect of CA on biofilm formation. SS2 incubated with sub-inhibitory concentrations of CA (1/4×, 1/8×, and 1/16× MIC) in a 96-well microplate for 96 h, washed by PBS for three times and fixed with methanol. The biofilm was stained with 1% crystal violet (wt/vol), then analyzed by microscopic observation and quantified by the absorbance at OD_595 nm_. (C and D). Effect of CA on biofilm eradication. SS2 preformed biofilms were subjected to CA exposure at different concentrations of 4×, 2×, and 1× MIC for 4 h, washed by PBS, and determined by the aforementioned methods as that in A and B. SS2 cultured in BHI medium was conducted as positive control. Magnification for morphological phenotypes observation was ×400. All assays were repeated three times with triplicate wells in one independent test. The significances of “*” and “**” indicated *P* < 0.05 and *P* < 0.01.

The crystal violet staining method was employed to assess the effect of CA on biofilm formation by SS2. A significant decrease in biofilm formation was observed in SS2 groups treated with CA at 1/4×, 1/8×, and 1/16× MIC in a dose-dependent manner, as illustrated in Fig. 3A. The absorbance value, which depicted the biofilm formation, in the CA treatment group (1/4 MIC) was reduced to nearly 50% as compared to the BHI control group (*P* < 0.01). The preformed biofilm eradication assay revealed the remarkable clearance of biofilms in the high doses of CA treatment at 1×, 2×, or 4× MIC (*P* < 0.01). A positive correlation between CA concentration and biofilm eradication was found, and more than 73% reduced biofilm was examined in the 4× MIC of the CA-treated group in Fig. 3C. These decreased quantification data were in accordance to the biofilm morphological phenotype observation results in Fig. 3B through D as shown by with loose, scattered, and less thick biofilm features in the CA treatment groups obtained from crystal violet staining analysis (Fig. 3B through D).

### Cinnamaldehyde inhibits the hemolytic activity and downregulates critical virulence factor expression of SS2

**Fig 4:**
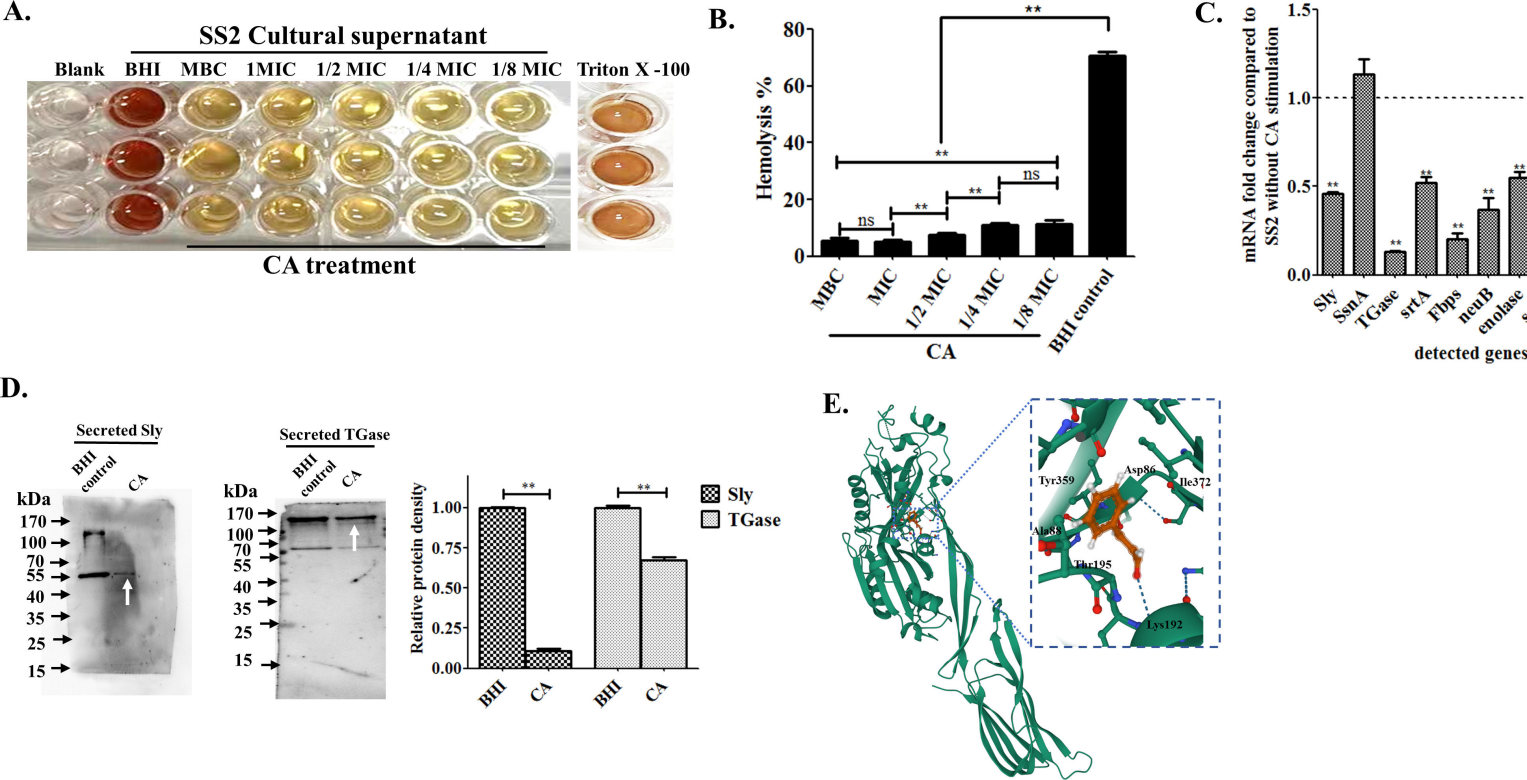
Cinnamaldehyde inhibits the hemolytic activity and downregulates virulence factors expression in SS2. (**A and B)** Effect of CA on hemolytic activity of SS2 tested in sheep blood cells. Equal volumes of SS2 supernatants after either incubation with CA at different concentrations or not were collected and added to 5% sheep red blood cells for 2 h at 37℃. Subsequently, 200 µL aliquots of the supernatants were carefully harvested and transferred into a 96-well polystyrene microplate to measure OD_540nm_. Erythrocytes treated with 0.2% Triton-100 served as 100% lysis control. The data were analyzed by triplicate repeated samples. (**C)** The mRNA fold changes of virulence-associated genes upon CA treatment (0.0625 µg/mL) in SS2 suspensions were determined by qPCR analysis. BHI-cultured SS2 without CA treatment was performed as a control. (**D)** Western blotting for Sly and TGase proteins. Overnight SS2 supernatant fractions treated with CA (0.0625 µg/mL) were prepared by the trichloroacetic acid/acetone precipitation method, and subjected to western blotting with further protein gray scale determination. The white arrows were indicated as targeted proteins. For B, C, and D, the signiﬁcant diﬀerences of “ns,” “*,” and “**” were indicated as *P* > 0.05, *P* < 0.05, and *P* < 0.01, respectively. (E) Molecular docking simulation of Sly and CA interactions. Probable interaction between CA and Sly (PDB ID: 3HVN) was predicted by the AutodockVina 1.2.2. The chemical structure of CA was acquired from PubChem, whereas the crystal structure of Sly was retrieved from the Protein Data Bank in the Swiss model.

The SS2 supernatant obtained from CA-treated groups showed a significantly impaired hemolytic phenotype in sheep blood cells, as demonstrated in Fig. 4A. Only 5.24%–11.48% erythrocyte lysis was found among the various concentrations of CA pre-incubated supernatants as compared to the BHI control (70.56%) with a significance value of *P* < 0.01, though no difference was indicated between the groups of 1/4× and 1/8× MIC (*P* > 0.05) (Fig. 4B). Real-time qPCR analysis was performed to evaluate the potential effect of CA on the crucial virulence gene expression in SS2. The targeted factors included the secretory suilysin gene of *Sly*, endonuclease A of s*snA*, transglutaminase of *TGase,* Sortase A of *srtA*, membrane-associated protein of *enolase*, fibronectin-binding protein of *Fbps*, sialic acid biosynthesis factor of *neuB*, as well as antioxidant factor of superoxide dismutase *sodA*, virulence transcription regulator *Rgg*, and orphan response regulator *covR* in SS2. With comparison to BHI control group, all the tested virulence or virulence-associated factors, except *sodA* and s*snA*, were subjected to notably reduced expression in the SS2 group treated with CA (*P* < 0.01) (Fig. 4C). The typical proteins of suilysin and TGase were further confirmed to remarkably decreased secretion in SS2 supernatant incubated with 1/4× MIC of CA by western blotting analysis (*P* < 0.01) (Fig. 4D). A molecular docking experiment was performed to determine the probable interaction between suilysin and the CA ligand. As illustrated in Fig. 4E, CA adopts a compact conformation to effectively bind to suilysin within the hydrophobic pocket. The 3D simulation models revealed an estimated binding energy of −5.33 kcal/mol for Sly-CA ligand interaction. This stable Sly-CA complex was formed possibly owing to the interactions that was facilitated by key amino acid residues of Lys192, Ile372, Asp86, K190, Ala88, and Thr195 within suilysin being in close proximity to the ligand CA.

### Cinnamaldehyde alleviates cytotoxicity induced by pathogenic SS2 strain and diminishes its adherence and intracellular viability in *in vitro* cells assays

**Fig 5:**
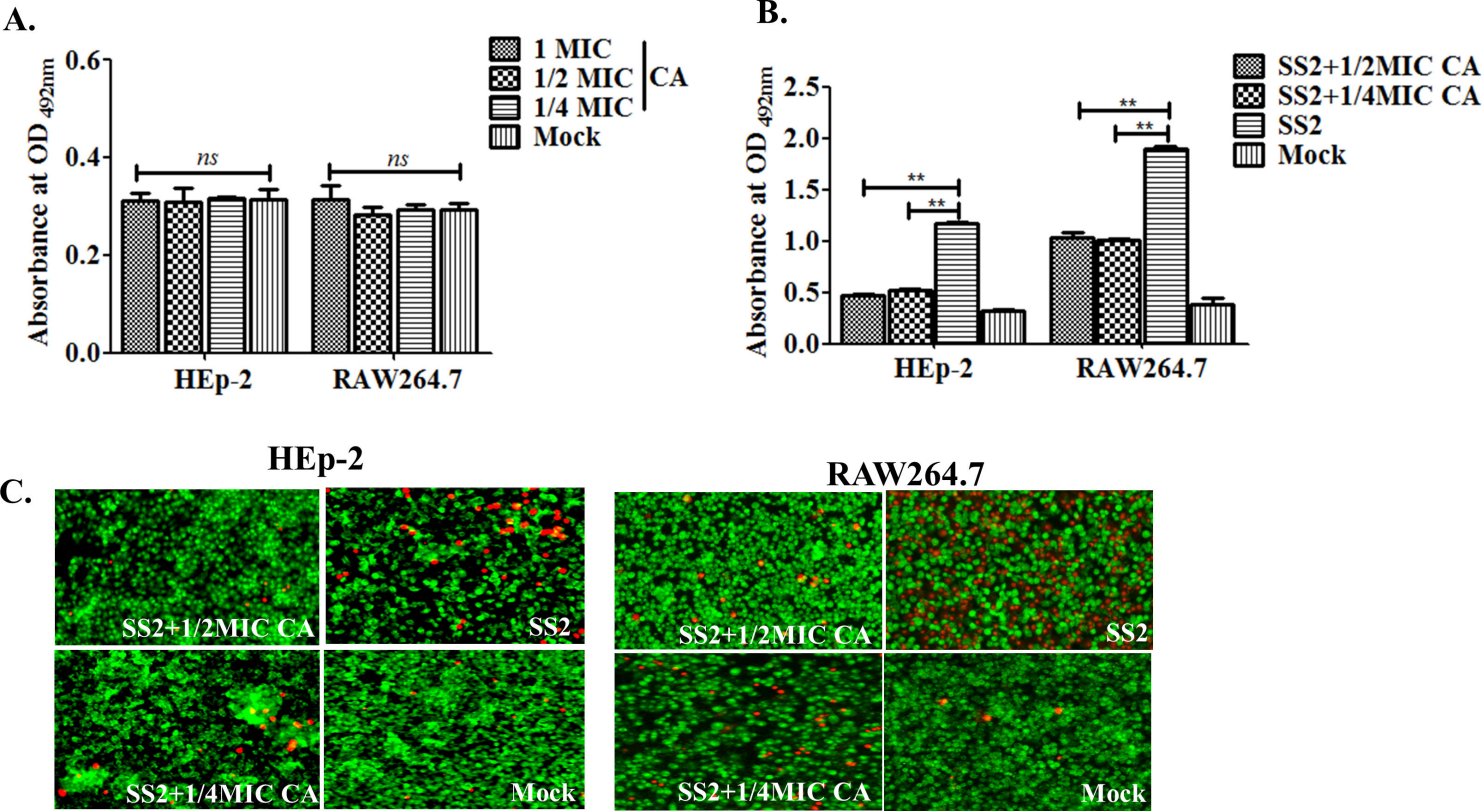
Cinnamaldehyde alleviates cytotoxicity induced by SS2 infection. (**A)** Evaluation of the intrinsic cytotoxicity of CA in different concentrations. (B and C) Effect of CA on cell cytotoxicity induced by SS2. Murine macrophage RAW264.7 and human laryngeal epithelial HEp-2 cells were used to examine the cytotoxicity induced by CA only in A, SS2 infection and the CA (1/2× and 1/4× MIC) cocultured SS2 infection groups in B. Cell cytotoxicity determination was detected by fluorescent observation in C after LIVE/DEAD staining and quantified by LDH activity kit in B. The live (green) and dead (red) cells were shown in C and the magnification for morphological observation was ×400. All assays were repeated three times with triplicate wells for A and B, and the significances of “ns,” “*,” and “**” were represented as *P* > 0.05, *P* <0.05, and *P* <0.01, respectively.

CA at different concentrations of 1×, 1/2×, and 1/4× MIC, were used to determine the safety in HEp-2 and RAW264.7 cells. As a result, the presence of CA alone at these three concentrations did not elicit cytotoxic effects on either HEp-2 or RAW264.7 for a 12 h incubation (*P* > 0.05) (Fig. 5A). Notably, the predominantly alleviated cytotoxicity induced by SS2 infection groups cocultured with CA at 1/2 or 1/4 MIC was shown by the intracellular LDH measurement in the HEp-2 or RAW264.7 cells in Fig. 5B (*P* < 0.01). Yet, the BHI control group of SS2 infection could prompt a direct host cell damage as observed in Fig. 5C. The quantification results of the cytotoxicity were in accordance to the phenotypes examined by fluorescence microscopy.

**Fig 6:**
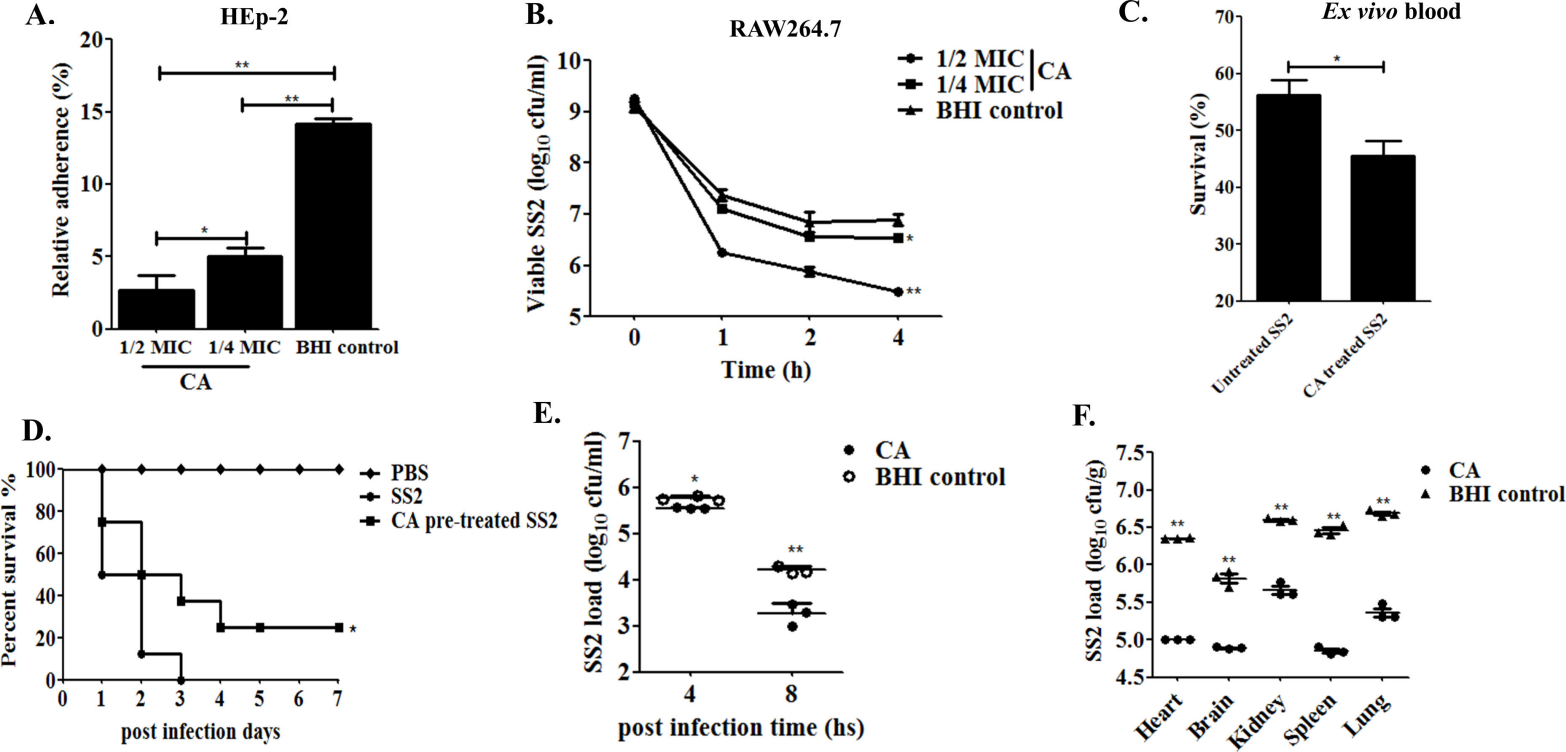
Cinnamaldehyde attenuates SS2 pathogenicity. (**A)** Adherence to HEp-2 cells. Equal volume of overnight CA (1/2× and 1/4× MIC) treated SS2 collection was added to HEp-2 in 24-well plates and incubated at 37°C for 3 h with 5% CO_2_. Relative adhesion rates were analyzed by counting the adhered SS2 colonies on BHI agar. (**B)** Intracellular viability in RAW264.7. The intracellular viability of SS2 in a different time within RAW264.7 cells was performed similarly to the adhesion assay except that SS2 adhered cells encountered another 1 h incubation with gentamicin (300 µg/mL) before the final lysis step. The phagocytosed viable SS2 was further counted and examined to assess the effect of CA on SS2 clearance by macrophages.** (C)** Survival in mouse whole blood. Overnight SS2 strain after coculturing with 1/4× MIC CA was subjected to clinically healthy mice whole blood incubation for 1 h at 37°C and viable SS2 in the mixture were counted by the serial dilution method. SS2 in BHI culture alone served as the control group. (**D)** The virulence evaluation of SS2 in a mouse infection model. After CA (0.0625 µg/mL) pre-incubation and washing by PBS, SS2 inoculates were intraperitoneally injected to eight mice in each group. Sterile PBS and SS2 without CA treatment were conducted as negative and positive controls. Clinical symptoms and mouse mortality were monitored and recorded everyday within 1 week post-infection. (**E)** SS2 dissemination among blood in the early infection course. The SS2 dissemination in orbital blood post 4 h and 8 h infection in three mice for each group encountered the aforementioned challenge as D. (**F)** Bacterial load of SS2 after CA treatment in mice organs. Viable bacteria load in mice organs collected from either CA-pre-incubated or SS2 cultured in BHI control group post 12 h infection. All these experiments were conducted three times and repeated in triplicate samples. “*” and “**” were indicated as *P* < 0.05 and *P* < 0.01, which was compared to the control group.

The influences of CA exposure on SS2 adhesion to the epithelial HEp-2 cells and phagocytosed SS2 viability within the RAW264.7 were investigated. In contrast with the SS2 in BHI-cultured control group, the CA-pretreated group of SS2 reduced its capability for adherence to the epithelial HEp-2 cells (*P* < 0.01) and diminished the viable CFU upon macrophagic cells killing effect (*P* < 0.05). The impact of CA for these two phenotypes was in a time- and concentration-dependent manner, and there was also a significant difference in efficacy between 1/2× and 1/4× MIC (*P* < 0.05) (Fig. 6A and B). Meanwhile, the suspension of SS2 obtained from cultures pre-incubated with CA at 1/4× MIC exhibited a highlighted susceptibility to immune clearance as shown by a reduced survival rate in *ex vivo* mouse whole blood assays (*P* < 0.05) (Fig. 6C).

### Cinnamaldehyde attenuates the virulence of SS2 and induces therapeutic potential for SS2 infection in a mouse model

Animal infection in BALB/c mice was used to evaluate the pathogenicity of CA-pretreated SS2 and therapeutic effects of CA against virulent SS2 infection. Firstly, the SS2 strain with an equivalent lethal dosage derived from cultures either treated with 1/4× MIC of CA or not were injected intraperitoneally into mice. The mice in the control group which suffered from SS2 infection, displayed significantly severe clinical signs such as ruffled hair, rough skin, blindness, movement disorders, and meningitis, and all died within 3 days post-infection. However, not only milder clinical symptoms with delayed lethal deaths but also improved survival rates (25.0%) in mice challenged with CA-pretreated SS2 strain were shown as Fig. 6D (*P* < 0.05). In addition, SS2 after CA treatment at 1/4× MIC could impede the pathogenic microbe dissemination in the bloodstream for the early post-infection time of 4 h (*P* < 0.05) and 8 h (*P* < 0.01) (Fig. 6E). The clinical sympton of retarding septicemia was verified by lower bacterial loads detected in the mice organs among the heart, brain, spleen, kidney, and lung in the CA-pretreated group (*P* < 0.01) (Fig. 6F).

**Fig 7:**
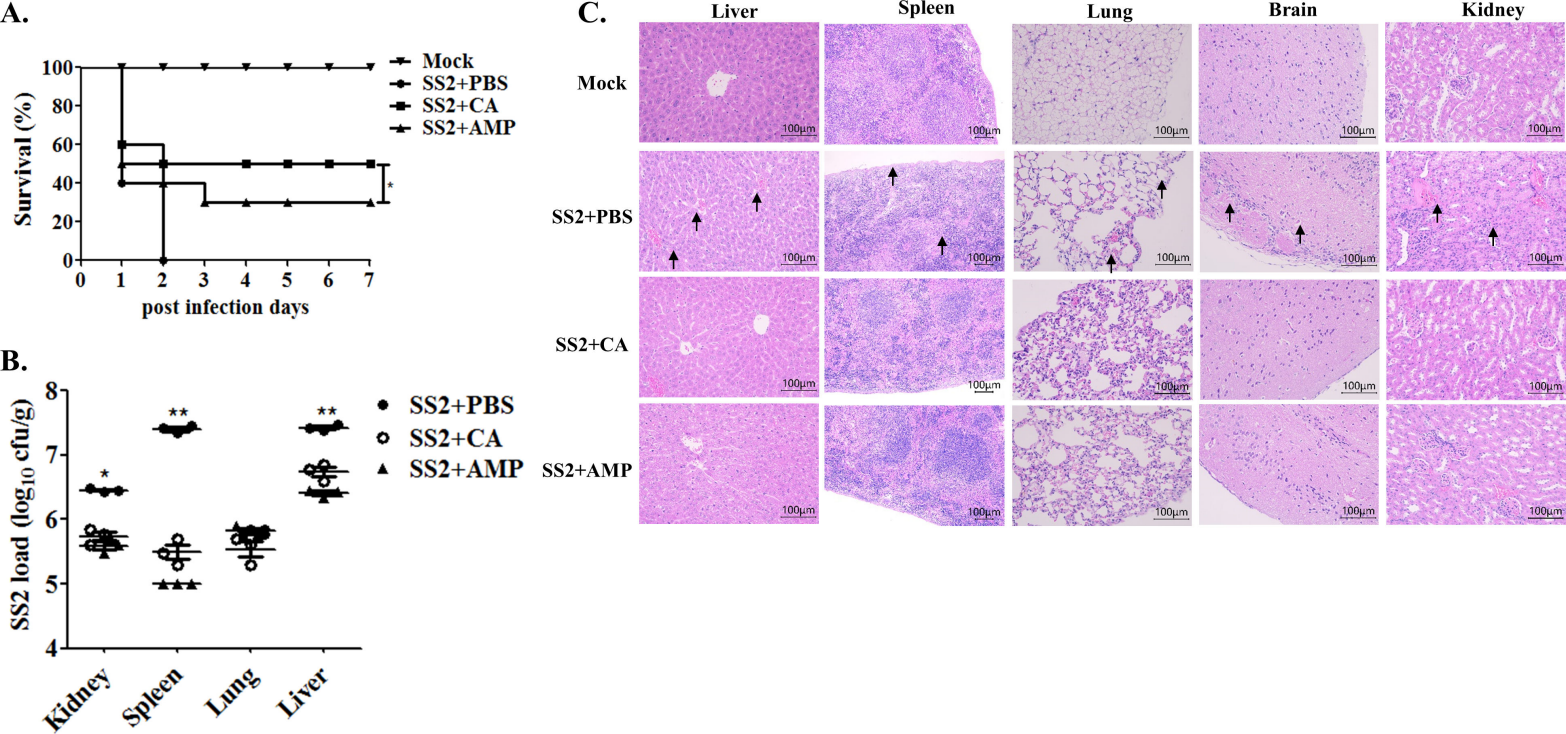
Potential therapeutic efficacy of cinnamaldehyde against SS2 infection in a mouse model. (**A)** Survival curves of mice in groups challenged with SS2 and then received the CA or conventional ampicillin therapy scheme. Pathogenic SS2 was pre-inoculated intraperitoneally into ten mice in each group for 2 h, administered CA (5 mg/kg), or conventional ampicillin (AMP, 5 mg/kg) treatment twice with an interval of 6 hs to anti-SS2 infection, and then monitored continuously for 7 days to depict the survival curves. No SS2 infection (Mock) and PBS-treated mice served as negative and positive control groups. (**B)** Bacterial load changes in mice organs treated with CA or AMP. For A and B, the experiments were conducted three times, and the significances of “*” and “**” were represented as *P* < 0.05 and *P* < 0.01, as compared with the AMP control group. (**C)** Histopathological lesion analysis upon microscopic observation. The black arrows indicated the lesion areas including cellular degeneration, inflammatory cell infiltration accompanied by hemorrhage in the liver and brain, wrinkling capsule and splenic corpuscles disintegration in the spleen, ruptured alveoli with hemorrhage and diffused inflammatory cell infiltration in the lung, and swelling glomerular with extravasated blood in the kidney.

*In vivo* therapeutic efficacy assays were performed using CA and a typical antibiotic of ampicillin as a parallel control against SS2 infection and evaluated in a mouse model. Result in Fig. 7A indicated that mice suffered from 2 h SS2 infection and then subjected to CA (5 mg/kg) treatment, displayed more subtle clinical symptoms with higher survival rate as 50% after 1 week when compared with that of PBS-treated SS2 infected mice (0%) (*P* < 0.01), and the ampicillin therapy group (30%) (*P* < 0.05). The significantly decreased bacterial burden in mice tissues, including the liver, spleen, and kidney, were expressed in Fig. 7B and manifested to be obviously weakened colonization in the SS2 infection group upon CA treatment on the basis of plate colony counting method (*P* < 0.01). Histopathological analysis from a microscopic perspective further demonstrated that the relieved inﬂammation and lethal damages, contrary to the SS2 alone and ampicillin-treated control groups, which revealed severe cellular degeneration, inflammatory cell infiltration accompanied by hemorrhage in the liver and brain, wrinkling capsule and splenic corpuscle disintegration in the spleen, ruptured alveoli with hemorrhage and diffused inflammatory cell infiltration in the lung, and swelling glomerular with extravasated blood in the kidney, were found in the CA therapeutic group against SS2 challenge (Fig. 7C).

## DISCUSSION

Widespread distribution of SS2 has aroused extensive attention for public health due to their zoonoses essence (45). In recent years, antimicrobial resistance phenotypes in SS2 have emerged and became even more worse in animal husbandry (9, 46). Herbal compounds and medicinal plants have emerged as promising therapeutic alternatives to mitigate AMR obsession (15). In the present study, cinnamaldehyde, a natural product derived from *Cinnamomum*, demonstrated effective antagonistic activity against SS2. MIC at 0.25 µg/mL and MBC at 0.5 µg/mL of CA were measured to exert definite growth inhibition and bactericidal activity to SS2 (Fig. 1). The inhibition and killing concentration values of CA for SS2 were lower than those reported for other pathogens that were also susceptible to this compound. The sub-inhibitory antimicrobial effect of CA, which showed no bacteriostatic efficacy for SS2, was determined as 1/4 MIC (0.0625 µg/mL). PAE, one of the typical indexes for pharmacodynamics, is being increasingly utilized in the formulation of antibiotic dosing regimens (32). The longer duration of PAE induced by an antimicrobial implies that a lower amount of this antibiotic is required for pathogen clearance (47). Surprisingly, as illustrated in Fig. 2A, the significantly prolonged PAE (over 7 h) induced by CA was found to be superior to that of tetracycline in SS2. This suggests that the promising application of CA may offer distinct advantages for the treatment of SS2 infections.

To explore the possible bactericidal mechanisms induced by CA, phenotypic alterations in SS2 cells following CA exposure were detected. In this context, it was discovered that the total protein synthesis in the SS2 suspension was interfered by CA co-cultivation at 1/4 MIC. Furthermore, CA demonstrated bacteriostatic activity against SS2 by disrupting the integrity of the cell membrane to enhance permeability, as evidenced by a significant increase in DNA and protein leakage to the supernatant, as depicted in Fig. 2B through D. Three-dimensional biofilms structures can act as arenas for microbial activities. The formation of biofilms typically commences when a cluster of microbes detect and adhere to a specific surface. As colonization advances, the production of the extracellular polysaccharide (EPS) matrix contributes to the solidification of the structure (48, 49). Biofilms have been shown to profoundly impact the efficacy of antimicrobial agents and the immune response, thereby contributing to AMR and facilitating the establishment of persistent/chronic infections (50). Interestingly, the effective inhibition for biofilm formation and elimination of preconstructed SS2 biofilm induced by CA at sub- and super-MIC, respectively, were tested and demonstrated in Fig. 3. These data verified the typical feature of CA which functioned the antimicrobial activity possibly owing to its increased cellular permeability and the inhibition of biofilm formation, and consistent to previous findings(25, 26, 51, 52).

The progress of pharmaceuticals targeting virulence factors has emerged as a significant and superior alternative to traditional antibiotics in combating infections caused by drug-resistant bacteria (53, 54). Suilysin, one of the critical virulence factors in SS2, is found to facilitate the colonization of host cells, evasion of host immunity, and induction of elevated inflammatory response, thereby playing important roles in meningitis and STSLS (55, 56). As previous studies reported on the natural medicinal molecules such as ellipticine hydrochloride and apigenin , the protein of Sly has emerged as a pivotal target accounted for the drug’s bactericidal efficacy (43, 44). Therefore, in this study, the hemolytic activity of SS2 upon CA treatment was analyzed. Likewise, CA could significantly decrease hemolysin release in SS2. The results for relative changes of mRNA and protein levels of Sly, as well as molecular docking simulations which predicted the spatial conformational binding, confirmed the aforementioned result and hinted the hypothesis that CA probable targets SS2 and suppresses sly expression. In addition, CA was found to downregulate the transcription of other crucial virulence-associated genes in SS2, including *TGase*, *srtA*, *enolase*, *Fbps*, *neuB*, *Rgg*, and *covR*. It was noteworthy that TGase, an important secretory anti-phagocytic factor of SS2 (57), exhibited a significantly reduced secretion in the CA-treated group for the first time (Fig. 4D).

To explore the impact of CA on SS2 pathogenicity to host, we carried out experiments using *in vitro* cell infection and a mouse infection model. The findings revealed that the CA could effectively reverse the cytotoxicity caused by SS2 infection in both epithelial HEp-2 and phagocytic RAW264.7 cells (Fig. 5). Moreover, treatment with CA significantly diminished the SS2 adhesion ability and rendered this pathogen more vulnerable to RAW264.7, thereby enhancing the clearance by mouse whole blood (Fig. 6). CA has been found to be absorbed and excreted in mice and humans without causing toxic side effects, and high doses of CA administered in mice do not lead to organ damage or weight loss, as previous studies have indicated (22, 58). Hence, a murine model was used to evaluate the changes in pathogenicity following CA treatment and its potential therapeutic efficacy against SS2 infection. The results revealed that the virulence of SS2 cells treated with 1/4 MIC of CA was significantly attenuated, resulting in milder clinical symptoms and improved survival rates among infected mice. Impeded dissemination of viable SS2, reduced reproduction, and lower bacterial load within the mice organs were shown in the CA-treated group (Fig. 6). Based on the methodologies clearly outlined (42, 44), a protective experiment for CA was performed in mice. The findings demonstrated that compared with the SS2-infected control group, the CA treatment in mice exhibited a 50% increase in survival rate, and this efficacy was superior to that of the traditional ampicillin therapy control group. Mice in the CA therapeutic group displayed significantly alleviated clinical-pathological phenotypes and notably reduced bacterial burden within organs, with considerable alleviation of tissue damage (Fig. 7). These findings strongly suggest that CA has the potential to effectively combat SS2 infections in mice.

In summary, this study demonstrated the credible antibacterial activity of CA with its therapeutic effect against *S. suis* type 2. The potential bactericidal effect of CA was attributed to its ability to modulate cell membrane permeability, impede bacterial protein synthesis, and prevent biofilm formation. Moreover, the reduced virulence of SS2 after CA treatment and its elevated protective efficacy against SS2 infection in mice were assessed. Future investigations are still needed to explore precisely the antibacterial properties of CA against SS2 clinical isolates and other *S. suis* serotypes, as well as the discrepant antimicrobial effects of other isomers or derivatives of CA on SS2, which deserved to be further examined.

## Data Availability

The authors validated that all data supporting the discoveries of this study were readily accessible in https://osf.io/sg9wh/?view_only=13f4a04ddb704b03992d7518040904c2.

## AFTER EPUB

[This article was published on 13 February 2025 with an error in the title. The title was corrected in the current version, posted on 6 March 2025.]
